# Comparison of yoga versus stretching for chronic low back pain: protocol for the Yoga Exercise Self-care (YES) trial

**DOI:** 10.1186/1745-6215-11-36

**Published:** 2010-03-31

**Authors:** Karen J Sherman, Daniel C Cherkin, Andrea J Cook, Rene J Hawkes, Richard A Deyo, Robert Wellman, Partap S Khalsa

**Affiliations:** 1 Group Health Research Institute, 1730 Minor Avenue, Suite 1600, Seattle, WA, USA; 2 Department of Epidemiology, University of Washington, Seattle, WA, USA; 3 Departments of Family Medicine and Health Services, University of Washington, Seattle, WA, USA; 4 Department of Biostatistics, University of Washington, Seattle, WA, USA; 5 Department of Family Medicine, Oregon Health and Science University, Portland, OR, USA; 6 Division of Extramural Research and Training, National Center for Complementary and Alternative Medicine, National Institutes of Health, Bethesda, MD, USA

## Abstract

**Background:**

Back pain, one of the most prevalent conditions afflicting American adults, is the leading reason for using complementary and alternative medicine (CAM) therapies. Yoga is an increasingly popular "mind-body" CAM therapy often used for relieving back pain and several small studies have found yoga effective for this condition. This study will assess whether yoga is effective for treating chronic low back pain compared with self care and exercise and will explore the mechanisms responsible for any observed benefits.

**Methods/Design:**

A total of 210 participants with low back pain lasting at least 3 months will be recruited from primary care clinics of a large healthcare system based in Seattle. They will be randomized in a 2:2:1 ratio to receive 12 weekly yoga classes, 12 weekly conventional therapeutic exercise classes of comparable physical exertion, or a self-care book. Interviewers masked to participants' treatment group will assess outcomes at baseline and 6, 12 and 26 weeks after randomization. Primary outcomes will be back-related dysfunction and symptom bothersomeness. In addition, data will be collected on physical measurements (e.g., flexion) at baseline and 12 weeks and saliva samples will be obtained at baseline, 6 and 12 weeks. Information will be collected on specific physical, psychological, and physiological factors to allow exploration of possible mechanisms of action through which yoga could relieve back pain and dysfunction. The effectiveness of yoga will be assessed using analysis of covariance (using general estimating equations - GEE) within an intention-to-treat context. If yoga is found effective, further analyses will explore whether yoga's benefits are attributable to physical, psychological and/or physiological factors.

**Conclusions:**

This study will provide the clearest evidence to date about the value of yoga as a therapeutic option for treating chronic back pain, and if the results are positive, will help focus future, more in-depth, research on the most promising potential mechanisms of action identified by this study.

**Trial registration:**

This trial is registered in ClinicalTrials.gov, with the ID number of *NCT00447668*.

## Background

Back pain is a common and costly health problem, with more than 50% of adults bothered by it each year [1] and 70% to 80% of adults afflicted by it at some time in their lives [2]. It is the most costly ailment of working age adults, with an estimated $33 billion spent annually on medical costs [3] and an estimated loss of $19.8 billion due to loss of worker productivity [4].

Although there are a wide variety of treatments for back pain, including medications, exercise, education, self-care, injections, life-style aids, manual therapies, complementary and alternative medicine (CAM) therapies, minimally invasive treatments and surgery, there is surprisingly little consistent evidence to support most of these treatments [5], due to unavailable, insufficient or conflicting evidence. However, low back pain patients are relatively dissatisfied with their medical care [6], especially in comparison to care provided by non-physician's [7-10]. Eisenberg [11] found that most people with back problems considered CAM care to be superior to conventional medical care for back pain.

### Yoga as a treatment for back pain

One of the most popular CAM therapies for back pain is yoga [12,13], (a mind-body intervention whose practice often includes physical exercise coupled with a focus on breathing to increase awareness. In 1997, an estimated 1.3 million Americans used yoga for relief of back pain, attending 9.1 million classes. According to national surveys, yoga practice has increased since then, with over 10 million Americans practicing yoga for health reasons in 2002 [14] and over 13 million in 2007 [15], although no published information indicates how many American practice yoga to relieve back pain.

In a review of 17 non-pharmacological therapies for low back pain that forms the basis of clinical practice guidelines issued by the American Pain Society and the American College of Physicians, Chou [16] found "fair evidence" (i.e., the strength of the evidence was limited by the number, quality, size, or consistency of the included evidence) that yoga is an effective treatment for this condition.

We found no systematic reviews of yoga for low back pain. However, six trials on this topic have been published [17-22], four of which suffered from very small samples, lack of a control group, high loss to follow-up, and/or only short-term follow-up (one week). One of the larger studies, with 28% drop-out from the yoga group, found yoga superior to standard care plus education at the end of 24 weeks of biweekly classes [22]. The most scientifically rigorous trial, with 101 participants, found yoga superior to the exercise and the self-care comparison groups by the end of the 12 week intervention period [19].

### Putative mechanisms for yoga's benefit on back pain

**Figure 1:**
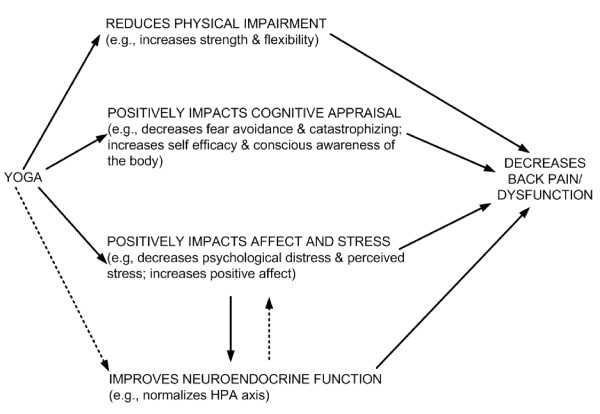
**Model describing possible mechanisms underlying the effectiveness of yoga for chronic low back pain**.

While the mechanisms of action responsible for yoga's potential positive effects on back pain patients are unclear, there are a number of plausible mechanisms, including physical movement, relief of physical and mental stress, and enhanced body awareness to reduce maladaptive movements and posture [19]. Figure 1 depicts a heuristic model, derived from the literature and comments from yoga teachers and participants in an earlier study of yoga [19]. The model includes several pathways by which yoga might improve back-related dysfunction or pain. The model hypothesizes that yoga may decrease the pain and/or dysfunction of persons with chronic back pain through positive effects on one or more of three major pathways: physical functioning of the back, cognitive appraisal about back pain, and general affect and stress. We further hypothesize that any positive effects of yoga on affect and stress will be associated with improvements in neuroendocrine stress mediators. These hypothetical pathways provide a framework for testing some of the most important potential mechanisms of action for yoga and for identifying which ones are most promising for future research. The hypothesized pathways by which yoga may lead to improved outcomes for persons with back pain are indicated in Figure 1 by solid arrows. The broken arrows represent some of the most important hypothesized secondary relationships. We consider model testing in this trial as exploratory with the primary goal of laying the groundwork for future studies that will more definitively elucidate yoga's mechanisms of action in the relief of back pain.

### Specific aims

To conduct a randomized clinical trial that will evaluate whether:

1. Yoga is an effective treatment for chronic back pain (i.e., superior to self care) in terms of improved function and reduced pain.

2. Yoga is superior to a conventional treatment ("therapeutic exercise"), which consists of similar amounts of stretching and gentle strengthening exercises, in terms of improved function and reduced pain.

If the benefits of yoga are confirmed, analyses will be performed to:

3. Identify specific psychological, physiological or physical factors that help explain (i.e., act as mediators) the beneficial effects of yoga.

The study hypotheses directly reflect the specific efficacy aims.

***Hypothesis 1: ***Yoga is more effective than self care. A finding that yoga is more effective than self care would confirm the results of smaller studies that yoga is a useful treatment for chronic back pain.

***Hypothesis 2: ***Yoga is more effective than conventional therapeutic exercise consisting of stretching and gentle strengthening exercises. A finding that yoga is more effective than stretching/strengthening exercises would suggest that yoga has additional benefits for back pain patients beyond physical movement per se.

If yoga is found superior to the self care and/or exercise control, we will investigate the mechanistic aim, which is more exploratory. The hypotheses are:

***Hypothesis 3: ***One or more of the "cognitive appraisal" variables (improvements in body awareness and/or self- efficacy, decreased fear avoidance) act as mediators of yoga's beneficial effects.

***Hypothesis 4: ***One or more of the "affect and stress" variables (improvements in psychological distress, perceived stress, and positive affect) act as mediators of yoga's beneficial effects.

***Hypothesis 5: ***One or more of the "physical function" variables act as mediators of yoga's beneficial effects.

***Hypothesis 6***: One or both of the "neuroendocrine function" variables (cortisol and DHEA) act as mediators of yoga's beneficial effects.

Identifying specific variables that act as mediators would help us understand better how yoga might actually exert its benefits.

## Methods/Design

### Overview

**Figure 2:**
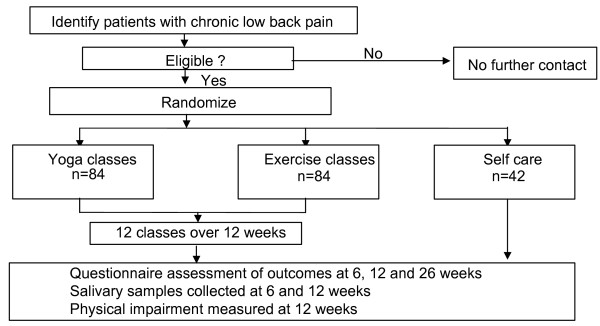
**Participant-focused flow chart**.

Approximately 210 persons with chronic low back pain will be randomized in a 2:2:1 ratio to receive 12 weekly 75-minute yoga classes, 12 weekly 75-minute conventional therapeutic exercise classes, or a self-care book (Figure 2). Back-related dysfunction and symptom bothersomeness, the primary outcomes, as well as potential psychological (cognitive and affective) mediators will be assessed at 6, 12, and 26 weeks post-randomization by telephone interviewers unaware of treatment assignment. Physical function (a physical mediator) will be assessed at 12 weeks post-randomization by a nurse practitioner unaware of treatment assignment. Finally, saliva samples will be collected at 6 and 12 weeks post-randomization to assay for cortisol and DHEA (dehydropiandrosterone), biomarkers that may be influenced by yoga practice. Statistical analyses will assess whether yoga is superior to exercise and/or a self-care book. If there is a benefit for yoga, additional analyses will explore potential mediating variables.

Bias will be minimized by a rigorous randomization procedure, by describing the study as one of three different "lifestyle approaches" to treating back pain, and by masking both interviewers and the nurse practitioner making physical measurements to the participant's treatment group.

### Study population

Participants will be recruited from Group Health Cooperative, a group-model, not-for-profit health care organization that serves over 400,000 members through its own primary care facilities in Western Washington. Group Health members with chronic low back pain of non-specific origin (as opposed to infectious, neoplastic, or inflammatory causes) will be eligible to participate.

### Inclusion and exclusion criteria

Health plan members from 20 through 64 years of age with ICD-9 diagnoses indicative of non-specific low back pain and whose pain has persisted at least three months will be eligible for the study if they rate their pain at least 3 on a 0 to 10 back pain bothersomeness scale and give informed consent. Non-specific low back pain was chosen as the condition for study because it is a common and expensive problem and a leading reason for the therapeutic use of yoga. Inclusion and exclusion criteria were developed to maximize the enrollment of appropriate patients while screening out patients who: have low back pain of a specific (e.g., spinal stenosis) or complicated (e.g., due to a medical condition) nature, for whom yoga or exercise is contraindicated (e.g., severe disk disease), or whose medical conditions might make it difficult or impossible to participate in the classes (e.g., gross obesity). These criteria are intended to exclude patients with medical conditions that might contribute to an increased risk of an adverse event, preclude fully informed consent (e.g., dementia), or lead to misinterpretation of the data (e.g., multiple sclerosis or diabetes with neurological symptoms that might interfere with pain sensation). To minimize the likelihood of including persons who have strongly preconceived notions about yoga or who wish to participate in this trial only to obtain free yoga classes that they were planning to pay for anyway, we will exclude potential participants who have practiced yoga for any reason within the past 3 months and for back pain within the past 12 months. Reasons for exclusion will be identified from automated data on ICD-9 diagnoses recorded during all visits over the previous year made by health plan members identified with low back pain-compatible ICD-9 diagnoses, telephone pre-eligibility interviews, and in-person visits. Tables 1 and 2 list the inclusion and exclusion criteria, the rationale for each criterion, and the source of information for each criterion.

**Table 1:** Inclusion Criteria

Inclusion Criteria	Rationale	Source*
Continuing member of Group Health Cooperative	Defined population that is easy to identify, recruit and follow-up	A, TI

20 through 64 years of age	Chronic low back pain in children results from different causes than those we are studying; older persons have higher risk of undiagnosed serious conditions causing low back pain	A

At least one primary care visit for back pain within the past 3-15 months	Efficient method for identifying people who may have chronic low back pain and who have been evaluated by a physician for their problem	A

Non-specific, uncomplicated low back pain, i.e., these ICD-9 codes: 724.2 Lumbago 724.5 Backache, unspecified 724.8 Other symptoms referable to back 846.0-9 Sprains and strains, sacroiliac 847.2 Sprains and strains, lumbar 847.3 Sprains and strains, sacral 847.9 Sprains and strains, unspecified site of the back	These codes are consistent with low back pain that is uncomplicated and specific in nature	A

Physician willing to have patients included in the study	Research policy	**

Lives within 45 minutes travel time from class location	Maximize attendance at classes	A

*A = Automated visit data

*TI = Telephone interview

** = determined by letter to physician before the sample identification

**Table 2:** Exclusion Criteria

Exclusion Criteria	Rationale	Source*
Low back pain has lasted < 3 months	Low back pain not chronic	TI

Bothersomeness of pain score of < 3	Back pain too mild to be able to detect improvement	TI

Abdominal Aneurism	Back pain due to, or possibly result of, specific	A
Cancer, metastatic	disease/condition	A, TI
Discitis		A
Disk disease		A
Fracture of vertebra		A, TI
Infectious cause of back pain		TI
Pregnancy		TI
Scoliosis, severe or progressive		A, TI
Spinal stenosis		A, TI
Spondylolisthesis		A, TI

Sciatica	Back problem of complicated nature, including medico-legal issues	TI
Seeking/receiving compensation/litigation for back pain		TI
Surgery, previous back, ever		TI

Blindness	Condition might make it difficult to attend the classes or practice at home	A
Deafness		A
Major Depression		IPE
No CD player for using home practice CDs		TI
Paralysis		A
Psychoses, major		A, TI
Schedules do not permit participation in classes or home practice (including planning to move out of town)		TI
Severe pain when bending or twisting spine		TI
Unable to walk two city blocks		TI
Unable to get up and down from floor		TI
Vision problems, severe		TI
Hearing problems, severe		TI
Lack of transportation		TI

Fibromyalgia, severe	Condition/circumstance might confound treatment	TI
Rheumatoid arthritis/Anklyosing spondylitis	effects or interpretation of data	A, TI
Other disabling chronic conditions (e.g., disabling heart or lung disease, diabetic neuropathy, receiving treatment for hepatitis)		TI
Planning on seeing a health care provider other than primary care provider for low back pain		TI

Dementia	Condition would make it difficult for fully informed consent	A
Unable to read or speak English		TI

Currently using exercise classes or yoga for back pain or has used one in past year; practiced yoga for any reason within past 3 months	Possible bias due to current or recent intervention users	TI

*A = Automated visit data

*IPE = In person examination

*TI = Telephone interview

Members determined to have any of the following during the telephone or in-person screening interviews will be excluded: non-specific causes or potential causes of low back pain (i.e., sciatica, underlying systemic or visceral disease, pregnancy, spondylolisthesis, spinal stenosis, cancer or unexplained weight loss, recent vertebral fracture); current use of yoga for their back pain or a back-specific exercise class or use in the past year; contraindication for yoga or exercise (e.g., severe disk disease); characteristics complicating the interpretation of findings (i.e., involved with litigation or compensation claim for back pain, evidence of severe or progressive neurologic deficits, previous back surgery, planning to seek other treatment for back pain, unstable medical or severe psychiatric conditions, major depression as determined by a score of 10 or greater on the Patient Health Questionnaire (PHQ)-8 [23]; inability to complete the study protocol (i.e., unable to speak or read English, plan to move out of town).

Recruitment procedures

Because the study intervention involves classes, we will recruit participants in seven cohorts consisting of 30 individuals each. We plan to recruit Group Health members who have made visits to their primary care physician for low back pain and whose pain has persisted for at least three months. However, if necessary to meet our recruitment goals, we will also post flyers in the Group Health primary care clinics and advertise in Group Health's quarterly magazine. Finally, in regions with fewer Group Health members, we may advertise the study to the general population.

Automated visit data will be used to identify Group Health members who have made visits to a primary care physician resulting in a diagnosis of low back pain. Those with diagnosis codes during the prior year corresponding to the exclusion criteria (e.g., paralysis) will be excluded. Primary care physicians will be given an opportunity to exclude *all *of their patients from receiving an invitation to participate in the study. Three months after their visit, potential participants will be mailed an invitation letter that explains the study and principal eligibility requirements, and invites participation. We will also include a reply card and a self-addressed, postage-paid envelope on which persons interested in the study can write their names and contact information and mail back to our study staff. An interviewer will then phone the members who respond to answer questions and determine provisional eligibility using a computer program that guides the interviewers through a series of screening questions. The screening process ends with documentation in a database of either ineligibility or provisional eligibility.

If provisionally eligible, the member will be invited to the clinic to confirm eligibility and obtain informed consent. After obtaining consent for the in-person examination, a trained study nurse will examine the patient to rule out sciatica, severe psychiatric conditions (i.e., a physician diagnosis of schizophrenia or bipolar disorder) and major depression (i.e., PHQ-8 score of more than 10) [23]. If confirmed eligible, a research specialist will guide the prospective participant through the consent form, including the elements required by the US Health Insurance Portability and Accountability Act. The study nurse will then obtain baseline physical measurements (Physical Impairment Inventory). After the physical examination, the participant will be taken to a nearby room where an interviewer will administer the baseline questionnaire using a computer assisted interviewing program.

### Randomization to treatment groups

After completing the baseline assessment, participants in each cohort will be randomized, in a 2:2:1 ratio, to the yoga, therapeutic exercise or self care groups. Treatment group will be automatically assigned by a computer-generated sequence of random numbers using a program that ensures that treatment allocation cannot be changed after randomization. Participants will be randomized in blocks of varying size to ensure balanced but unpredictable assignment of participants to all groups. The sequence of randomization and blocking factor will differ for each cohort. All participants will be given the saliva collection kits and instructions for collecting the baseline saliva samples. Participants randomized to the yoga or exercise classes will be given a sheet of paper with their class time, study contact information and a map showing their class location. Just prior to the first class, participants randomized to yoga or exercise will be given a reminder call of their class time. Participants randomized to the self care group will receive a book about self-management of back pain (The Back Pain Helpbook[24]).

### Study treatments

Participants will be randomized to yoga classes, therapeutic exercise classes or receipt of a self-care book. Both the yoga and exercise interventions will consist of 12 weekly 75-minute classes supplemented by home practice. The dose of yoga in this study was chosen because it was found effective in our previous trial back pain [19]. Conceivably, the "optimal dose" of yoga could differ from the amount of yoga we will provide, but we are unaware of studies that have examined this issue. Participants will be asked to report their weekly home practice on pre-printed home practice logs. For legal and ethical reasons, all participants will continue to have access to the services normally available under their health insurance plan, regardless of the intervention they are assigned. The treatments are described below.

#### A. Yoga

We will use viniyoga, a therapeutically-oriented style of yoga that emphasizes safety, is easy to learn, and has rigorous teacher training standards, as the basis of our intervention. Because viniyoga focuses on the purpose of each posture, rather than on its precise form, it tailors the postures to abilities of each individual's body. This emphasis on safe performance of individual poses and careful sequencing of the poses minimizes the risk of injury and discomfort. Viniyoga also emphasizes special breathing techniques that reduce stress and increase awareness of the body, considered important for reducing injury [21].

Our yoga intervention ("Yoga for Backs") [19] includes six yoga posture sequences created using the principles of viniyoga [25] and designed for people with low back pain who have no previous experience with yoga. The postures for each class were selected, with minor adaptations, from a core of 17 relatively simple postures (Table 3). Each posture sequence will be repeated in two successive classes with every other class having a different focus (i.e., relaxation; developing strength, flexibility and large muscle movement; asymmetrical postures; strengthening the hip muscles; lateral bending; integration and customizing a personal practice). All classes will emphasize the use of postures and breathing for managing low back symptoms.

**Table 3:** Content of Each Yoga Class

	Class Number					
**Yoga Class Components**	**1 and 2**	**3 and 4**	**5 and 6**	**7 and 8**	**9 and 10**	**11 and 12**

Introductory Breathing Exercise	x	x	x	x	x	x

Cobra posture and variations	x	x	x	x	x	x

Knee to chest and variations	x	x	x	x	x	x

"Wheel" posture variations	x	x	x	x	x	x

Bridge posture variations	x	x		x		x

Supine butterfly posture	x	x	x	x		

Extended leg with variations		x	x	x	x	x

Warrior posture variations		x				x

Standing forward bend			x		x	x

Kneeling forward bend with variations		x	x		x	

Chair pose		x	x			x

Lying twist/lying lateral		x			x	x

Swimmer's posture with variations			x		x	x

Extended side stretch			x			

Lunge				x		

Lying side hip strengtheners				x		

Kneeling lateral posture					x	

Standing lateral posture					x	

Deep Relaxation	x	x	x	x	x	x

Final Breathing Exercise	x	x	x	x	x	x

Each class will include an initial breathing exercise, a sequence of 5 to 11 postures, a guided deep relaxation and a final breathing exercise. Most postures will not be held for prolonged periods but will be repeated 3 or 6 times sequentially in a flow. In addition to the introduction of new poses in each new sequence, many poses and basic concepts will be repeated throughout the series to facilitate and encourage home practice. Participants will be asked to practice every day for 20 minutes to maximize the benefit of the intervention. At the end of the 1^st^, 4^th^, 7^th^, 9^th ^and 11^th ^classes, they will receive a printed handout outlining the sequence of poses and a corresponding CD to be used for their home practice. The CD will assist them in practicing the postures with the appropriate mental focus.

#### B. Conventional therapeutic exercise

The exercise intervention will be identical to the yoga classes in terms of length of classes (75 minutes), number of classes (12) and amount of physical exertion required (stretching and strengthening exercises only, with no extreme movement). Most of the class will involve conventional stretching exercises that are appropriate for patients with chronic back pain, including a comprehensive set of exercises that stretch all the major muscle groups, with an emphasis on the trunk and legs. The intervention will include all 12 stretching exercises used in the exercise intervention of a previous study [19] (i.e., gastrocnemius, soleus, quadriceps, posterior and inferior shoulder, upper trapezius, hip flexor, back extension, back rotation, hamstrings, hip external rotators, back flexion), plus 3 additional stretches (hip internal rotators, hip adductors and hip flexion). Each stretching exercise will be held for approximately 60 seconds and repeated once. In addition to a complete set (15) of full-body stretches, the class will begin with a five minute warm-up period consisting of basic aerobics steps (i.e., one minute each of walking in place, marching, lateral shuffling, turning and reaching, and box step) and will also include four exercises from our previous study that strengthen the back, abdomen and hips (i.e., squats, crunches, oblique crunches, back extensions) [19]. Over the 12-weeks of class, the number of weekly repetitions of each strength exercise will be increased from 8 to 30 in increments of two. Specific strength exercises will be practiced in separate sets of 5 to 10 repetitions. For example, if 16 repetitions of all 10 of the strength exercises are to be performed, 8 repetitions of each of the 10 exercises will first be performed followed by another 8 repetitions. Participants will be asked to practice every day for 20 minutes to maximize the benefit of the intervention. Printed handouts will be provided to facilitate practice. In addition, a DVD demonstrating all the exercises will be provided to assist participants in practicing safely.

#### C. Self-care book

Participants in this group will be mailed a copy of *The Back Pain Helpbook*, a 224-page evidence-based book on self-care for back pain. The book includes information on causes of back pain, initiating a comprehensive fitness and strength program, and appropriate life-style modification and guidelines for managing flare-ups [24]. We believe that providing these materials may mitigate the disappointment among those randomized to "continued usual care". Such disappointment could conceivably bias reports of symptom severity and dysfunction and result in higher rates of loss to follow-up. Sending these participants a book also allows us to describe our study as a comparison of different lifestyle interventions, which helps create more uniform expectations for the different interventions.

#### Class sites

The yoga and therapeutic exercise classes will be offered in large, quiet classrooms at Group Health facilities that are centrally located and provide easy access for persons with physical limitations.

#### Class instructors

All our yoga instructors' will be certified in viniyoga, which has one of the most comprehensive yoga teacher training programs in the West. Training takes at least three years to complete and leads to certification at the highest level (500 hours) by the Yoga Alliance, the group that has established voluntary national standards for yoga teachers. All certified viniyoga instructors are trained to work with people suffering from back pain. Ms. Robin Rothenberg, our local yoga consultant who helped develop the intervention for our previous study [19], will help us select qualified instructors and will train them to the protocol.

We will require our exercise class instructors to be licensed physical therapists who have previous experience teaching groups. John Maisano, the physical therapist who developed our "Healthy Backs, Healthy Lives" exercise class series and who will revise the exercise intervention for this study, will help us select qualified instructors and will train them to the protocol.

### Training and monitoring of class instructors

All of the yoga and exercise instructors will need to agree to adhere strictly to the treatment protocol and to complete our training programs for yoga (to be taught by trial Principal Investigator Sherman and yoga expert Ms. Rothenberg) and exercise (to be taught by Dr. Sherman and exercise expert, Mr. Maisano). This training will include a thorough discussion of the class protocols for each intervention. There will be an opportunity for each instructor to practice leading a class as part of the training and to discuss any concerns they may have.

During the class series, Dr. Sherman will be in weekly contact with the instructors and will inquire about positive experiences, adverse events, concerns raised by participants, ability to stay within the protocol, and any other questions that may arise. Research specialists will attend each of the classes to collect home practice information and take attendance. Dr. Sherman will also attend at least one class in each series to ensure that instructors are adhering to the protocol.

### Assessment of outcomes

We will collect data on sociodemographic characteristics, back pain history, back outcome measures, treatment-related information, potential confounders, co-inventions, and a number of key psychological, physical and physiological factors that we hypothesized might mediate any effects of yoga on chronic low back pain (Figure 1). We will measure short-term (6-week and 12-week) outcomes to determine whether the classes have had any benefit prior to or immediately following their completion, and longer-term (26-week) outcomes to determine if such benefits persist and if participants are continuing to practice their new skills. These measures are summarized in Table 4, with the most important measures described in the text that follows.

**Table 4:** Content of baseline and follow-up assessments

Measures	Baseline	6-Wk	12-Wk	26-Wk
**BASELINE INFORMATION**				

Sociodemographic characteristics	x			

Back pain history	x			

Expectations of treatment	x			

Knowledge of yoga and therapeutic exercises	x			

**CORE SET OF OUTCOMES FOR BACK PAIN STUDIES**				

* Roland Morris Disability Questionnaire (RDQ)(dysfunction)	x	x	x	x

* Bothersomeness of low back pain	x	x	x	x

Satisfaction with back care	x	x	x	

Disability days	x	x	x	x

Patient global rating of improvement		x	x	x

**TREATMENT-RELATED INFORMATION**				

Adverse experiences		x	x	x

Perceptions of yoga and therapeutic exercise classes, including instructors			x	

Adherence to assigned treatment, including home practice		x	x	x

**POTENTIAL CONFOUNDERS**				

Use of co-interventions: medications	x	x	x	x

Use of co-interventions: other treatments			x	x

Daily exercise and job-related activity	x	x	x	x

Smoking status	x			x

Lawsuits, workers compensation (initial exclusion)			x	x

Body Mass Index	x			x

**POTENTIAL MEDIATING VARIABLES**				

***Positively impacts cognitive appraisal***				

Fear avoidance	x	x	x	x

Self-efficacy	x	x	x	x

Conscious awareness of body	x	x	x	x

***Positively impacts affect and stress***				

Psychological distress	x	x	x	x

Perceived stress	x	x	x	x

Positive emotions	x	x	x	x

***Reduces physical impairment***				

Physical Impairment Index	x		x	

***Improves neuroendocrine function***				

Salivary cortisol	x	x	x	

Salivary DHEA	x	x	x	

* Co-primary outcome measures

A core set of outcome measures, covering five domains (back-related function, pain, general health status, work disability and patient satisfaction) has been recommended in a general review of outcomes assessments for evaluating treatments of spinal disorders [26]. Our choice of outcome measures closely follows these recommendations.

#### A. Primary outcome measures

The modified Roland-Morris Disability Questionnaire (RDQ) will be used to measure back-related patient dysfunction [27]. This instrument, which asks 23 yes/no questions selected because of their relevance for patients with back problems, takes approximately five minutes to complete [26]. The RDQ is one of the two most popular instruments used by back pain researchers for measuring function [28], has been found to be reliable, valid and sensitive to clinical changes [27,29-34], and is well suited for telephone administration [28].

Because there are individuals who are very bothered by even a small amount of pain and others who are not bothered by even moderate pain, we felt that the important outcome was not so much a difficult-to-interpret report of pain severity as is often used, but the extent to which participants' lives are affected by whatever level of pain they felt (i.e., how bothered they are by their symptoms). We therefore have chosen as our primary measure of symptoms a 0 to 10 scale of "symptom bothersomeness", where 0 represents "not at all bothersome" and 10 "extremely bothersome". Participants will be asked at baseline and during all follow-up interviews to rate how "bothersome" their back-pain has been during the previous week. This question has worked well in our previous studies with both mailed and telephone interviews [27,35,36]((and appears to have substantial construct validity, i.e., is highly correlated with measures of function and other outcome measures [37].

#### B. Potential mediating variables

We will measure a number of psychological, physical and physiological variables that our heuristic model (Figure 1) hypothesized could mediate any effects of yoga on low back pain. If yoga is superior to self care and/or to exercise, these will be included in analyses designed to address Aim 3.

##### a. Cognitive appraisal

Aspects of cognitive appraisal will be assessed with measures of fear avoidance, self-efficacy, and self-awareness.

*Fear avoidance *will be measured with the Tampa Scale for Kinesiophobia [38], a 17-item scale measuring back pain patients' fears of movement, exercise and serious underlying disease. We will use 10 of the 17 items from this scale, eliminating several items found confusing to participants or redundant [39]. This 10-item version retains acceptable internal consistency (Cronbach's alpha = 0.76), is easier and quicker to administer than the full form and has proved sensitive in detecting intervention effects in clinical trials [39].

We will measure *self-efficacy *using 5 of 8-items of the Arthritis Self-efficacy Scale [40], modified for back pain patients. This scale has been found to be both valid and reliable [40].

We will measure *awareness *specifically as conscious awareness of the body using two complementary and validated questionnaires, the Body Awareness Questionnaire [41] and the Body Responsiveness Questionnaire [42]. The Body Awareness Questionnaire contains 18-items that measure self-reported attentiveness to normal non-emotive bodily processes, including sensitivity to body cycles and rhythms, ability to detect small changes in normal function, and ability to anticipate bodily reactions. It has been found to have good internal consistency and test-retest reliability [41,42]. (The Body Responsiveness Questionnaire, a 7-item scale designed to measure responsiveness to bodily sensations, also has good internal consistency (Cronbach's alpha = 0.83) [42].

##### b. Affect and stress

We will measure psychological distress, perceived stress and positive states of mind.

*Psychological distress *will be measured with the 5-item Mental Health Index of the SF-36 [43]. This scale, which assesses general mental health, including depression, anxiety, behavioral-emotional control, and general positive affect, is brief and reliable and has shown good agreement with more comprehensive measures of mental health [44].

*Perceived stress *will be measured with the 10-item version of the Perceived Stress Scale [45], the most widely used self-report measure of psychological stress.

We will measure *positive states of mind *with the Positive States of Mind Scale, a 6-item scale that has good internal consistency (Cronbach's alpha ranges from 0.65 to 0.77) [46,47] and is inversely related to anxiety and to indicators of stress [46].

##### c. Physical impairment

We will measure physical impairment using Waddell's Physical Impairment Index, which includes the results of 7 physical tests (total flexion, total extension, lateral flexion, average straight leg raise, and bilateral straight leg raise, all measured in degrees; and spinal tenderness and sit-up, both measured as yes/no) [47]. The Index includes measures of both flexibility and strength. We will also be able to use each test as an independent measure of physical impairment.

##### d. Biomarkers of neuroendocrine function

We will measure *cortisol *and *DHEA *from saliva samples. Salivary hormones provide an unobtrusive way to sample in naturalistic settings. Salivary cortisol and DHEA are highly correlated with serum levels [48,49]. Saliva samples will be collected from each participant at wakening, 30 minutes after wakening, and bedtime over a two-day period (6 samples total) at baseline, 6 and 12 weeks. Cortisol levels will be measured at all 6 time points, while DHEA will be measured only at wakening and bedtime. The saliva samples will be analyzed using enzyme immunoassays for cortisol and DHEA at the STAR (Saliva Testing and Reference) Laboratory, Seattle WA, a CLIA certified laboratory specializing in the analysis of saliva samples.

### Data collection and quality control

We will collect information on the outcomes at every stage of our recruitment, randomization, and treatment processes so that we can report patient flow according to the CONSORT guidelines [50]. Specifically, we will record the number of invitation letters sent, the number of responses received, the resolution of these responses (i.e., ineligible, refused, eligible and randomized, other), the number of participants assigned to each class who actually attended classes and the number of classes they attended, the number of participants in each treatment group providing each type of follow-up data at each follow-up (e.g., questionnaire, saliva samples), the number of withdrawals due to perceived ineffectiveness of the intervention, adverse experiences, loss to follow-up or other causes, and the number of participants completing the trial.

We will implement procedures to ensure that randomization is proceeding as planned, recruitment is on schedule, data collection forms are accurately entered into databases, the computer assisted telephone interviewing (CATI) system is storing data correctly and that data can be accurately transferred and retrieved as needed. We will develop a relational database to track information on every stage of recruitment, randomization, class attendance, and outcomes assessment so we can use standard automated reports of patient flow. All data system processes will be thoroughly tested prior to the start of recruitment.

The CATI programs will contain range and logic checks. Participant attendance information collected during the classes will be double key entered into a database that also contains logic checks. Prior to recruitment, all data systems will be tested with imaginary participants. Data will be examined for completeness using computer programs developed specifically for that purpose. In addition, we will test all analytic programs to ensure that the analyses are accurate.

Saliva samples will be labeled only with unique serial numbers and stored in a secure -70°C freezer until they are assayed at the STAR (Saliva Testing and Reference) laboratory. Samples will be taken to the STAR laboratory periodically in special shipping containers with dry ice to ensure they remain frozen. Standard quality control procedures will be used to ensure assay integrity, including testing all specimens in duplicate wells and using both manufacturer and in-house quality control samples to measure inter-and intra-assay coefficients of variance. Individual samples that exceed a 12% coefficient of variance will be rerun to ensure accurate concentrations are calculated.

In addition, at both Group Health and the STAR laboratory, saliva specimens will be handled and processed only by staff members who are trained and certified by each institution to receive and/or ship biospecimens and who will agree to comply with all institutional policies and procedures to ensure safe handling of the specimens.

To maintain the confidentiality of patient-related information in the database, unique participant study numbers will be used to identify data on treatment and patient outcomes. The password security system will assign appropriate levels of computer privileges to different groups of database users. This will ensure that all masked personnel remain masked to treatment group.

Computer files with participant names will be password protected with access restricted to staff using this information to recruit participants, contact class participants or obtain follow-up data, and interact with any patients reporting adverse events. Any paper data forms, such as home practice logs and saliva logs, will be identifiable only by unique study identification numbers and kept in locked filing cabinets. Finally, all analysis data files will be password protected. Full data back-up procedures will be performed nightly, with partial data back-up throughout the day.

### Protection of human subjects and assessment of safety

#### A. Protection of human subjects

This study was approved by the Human Subjects Review Committee (IRB Number 00000668), which serves as the institutional review board or ethics committee, for Group Health Cooperative. The study will be carried out in compliance with the Helsinki Declaration.

#### B. Safety monitoring

Given the favorable safety profile from previous studies of yoga, a primary care physician experienced in exercise research, independent of the study team, will monitor the safety of participants in this trial. To protect the safety of the study participants, the physician monitor will evaluate new data on adverse-experiences we will provide every six months during the trial. Based on the observed adverse effects of yoga and exercise, the physician montior will make recommendations on a regular basis to the study Principal Investigator and the Office of Clinical and Regulatory Affairs at the National Center for Complementary and Alternative Medicine (NCCAM) regarding continuation, termination, or other modifications of the trial.

#### C. Adverse events

Participants will be asked about adverse experiences at each class and during the 6, 12, and 26-week telephone interviews. We will define an adverse experience as any unfavorable and unintended sign, symptom or disease temporally associated with the use of yoga or therapeutic exercises that could reasonably be related to the interventions. If a participant develops a Serious Adverse Experience (i.e., any adverse event occurring during treatment that results in any of the following outcomes: death, a life-threatening adverse event, inpatient hospitalization or prolongation of existing hospitalization, a persistent or significant disability/incapacity, a congenital anomaly/birth defect, or cancer), it will be promptly (within 7 days) reported to the physician monitor, and if it is at least possibly due to yoga or therapeutic exercise, it will also be promptly reported to the Group Health Human Subjects Review Committee and to NCCAM's Office of Clinical and Regulatory Affairs. In addition, all adverse experiences in the trial will be summarized in routine reports to the physician monitor and the IRB.

#### D. Stopping rules

No formal stopping rules will be established in advance for efficacy. The trial will be stopped only if the physician monitor believes there is an unacceptable risk of serious adverse events in one or more of the treatment arms. In this case, the physician monitor could recommend terminating one of the arms of the trial or the entire trial.

### Statistical issues

#### A. Sample size and the detectable difference

Because patient function (or dysfunction) is generally considered the more consequential of our two primary outcome measures [27] (the other being bothersomeness of back pain), sample size calculations are based on the Roland-Morris Disability Questionnaire (RDQ). Our sample size is designed to ensure that we have good power to detect a clinically significant difference of 2.5 points for pairwise comparisons (yoga to self care and yoga to exercise) on the RDQ (Aim 1 and 2) and adequate power to detect a difference of 1.7 points between the yoga and exercise groups that would be of interest when exploring mechanisms of action (Aim 3). We think that a smaller detectable difference between yoga and exercise can be justified when examining mechanisms of action because in the Aim 3 comparison of yoga and exercise, we focus on the *additional *benefits of yoga compared with exercise, anticipating that a portion of yoga's clinical effects would actually result from movement.

We have accommodated these dual power needs by proposing a 2:2:1 randomization ratio (yoga: exercise: self care), with a total of 210 participants. Assuming 10% loss to follow-up (which is slightly higher than in our previous study [19]), there would be outcomes data for 75:75:38 participants in the yoga, exercise, and self care groups, respectively. To protect against multiple comparisons, we will use Fisher's protected least significant difference approach, which has been shown to have desirable properties when there are three groups [51]. This approach makes pair-wise comparisons between the three treatment groups only if the overall *F*-test is significant. The power of this omnibus *F*-test depends on how the means from the three treatment groups differ. We therefore assumed that the yoga group would be 1.7 RDQ points superior to the exercise group, which would, in turn, be 0.8 RDQ points better than the self care group (giving a difference of 2.5 points between the yoga and self care groups). We chose a 1.7-point difference between yoga and exercise because it is slightly more conservative (i.e., smaller) than that found in our previous study [19].

Our estimates of the standard deviations of our primary outcome measures adjusted for pre-randomization baseline values were derived from analyses of covariance of 12-week follow-up data estimated from the 101 study participants in our 3-arm pilot study: RDQ standard deviation (SD) = 3.68 and bothersomeness SD = 2.38. With our proposed sample size, the omnibus *F*-test for the RDQ score will have 92% power for detecting a statistically significant difference among the three treatment means with the distribution assumed above. If this omnibus test isstatistically significant we will address Aims 1 and 2 of the study by comparing the appropriate pairs of means, as discussed below. To detect a pair-wise difference of 2.5 RDQ points, we will have 92% power for the yoga (or exercise) to self care comparison and 98% power for the yoga to exercise comparison. For Aim 3 we will have 92% to detect a pairwise difference of 2.5 RDQ points between yoga (or exercise) and self care and 80% power to detect a pairwise difference of 1.7 points between yoga and exercise.

Our sample size will also provide adequate power to detect a clinically important difference of 1.5 on our 0 to 10 bothersomeness measure. For the omnibus *F*-test, we will have 89% power for detecting a significant difference of 1.5 points among the three groups (if we assume a difference of 1.1 points between the yoga and exercise groups and of 0.4 points between exercise and self care). For a difference of 1.5 points on the bothersomeness measure, we will have 88% power for the yoga (or exercise) to self care comparison and 97% power to for the yoga to exercise comparison. For Aim 3, we will have 88% power to detect a pairwise difference of 1.5 bothersomeness points between yoga (or exercise) and self care and 80% power to detect a pairwise difference of 1.1 points between yoga and exercise.

At each time point, both primary outcomes (function and symptoms) will be tested at the 0.05 level because they address separate scientific questions. Analyses of both outcomes at all follow-up times will be reported, imposing a more stringent requirement than simply reporting a sole significant outcome. Arguments against adjusting for multiple comparisons in this situation have been made by Rothman [52,53] and others [54].

The power calculations are based on simple comparisons of the follow-up scores at a single point in time with adjustment for baseline values using analysis of covariance. We also plan to adjust for other baseline characteristics (e.g., age, gender, and baseline covariates found predictive of 10-week outcomes). Inclusion of such baseline covariates can improve precision of the variance estimate and therefore increase power.

Since assessment of Aim 3, the mediator analysis, is dependent on the results from Aim 1 and Aim 2, the study was not directly powered for Aim 3. We ran a simple power analyses for the primary mediator of interest, body awareness, assuming the expected sample sizes of 75:75:38, a single time point, and the RDQ Score as the outcome. We found a median power of 0.86 to detect body awareness as a significant mediator for yoga compared to self care and a median power of 0.83 for to detect body awareness as a significant mediator for yoga compared to exercise.

In summary, we have excellent power to detect a clinically meaningful difference on the omnibus test and the pairwise comparisons with self care for Aims 1 and 2 as well as adequate power to evaluate the yoga - exercise comparison for the mediator analysis for Aim 3. Although powered to detect a clinically significant difference on the RDQ, the resultant sample sizes will provide ample power for both of our primary outcome measures.

#### B. Statistical analysis

We will analyze outcomes from all follow-up time points in a single model, adjusting for possible correlation within individuals using generalized estimating equations (GEE) [55]. Because we cannot reasonably make an assumption of constant or linear group differences over time, we will include an interaction between treatment group and time point. In this case, the multivariate model that includes all time points provides very similar results to fitting separate analysis of covariance models over time and should not substantially influence statistical power.

We also plan to adjust for other baseline characteristics. Specifically, gender, age, pain traveling below the knee but not meeting the criteria for sciatica, job related activity, and Body Mass Index. In a randomized trial of this size, most baseline values and other covariates are unlikely to differ between randomized groups. However, inclusion of baseline covariates can improve the precision of the estimate and therefore increase power to detect differences.

We will use an intent-to-treat approach in all analyses, *i.e*., individuals will be analyzed by randomized group regardless of participation in any classes. This minimizes biases that often occur when participants not receiving assigned treatments are excluded from the analyses. The linear regression model (analysis of covariance) we will use is of the form:

where *Y*(*t*) is the response at follow-up time *t*, *Baseline *is the pre-randomization value of the outcome measure, *Trt *includes dummy variables for the yoga and exercise groups, *Time *is a series of dummy variables indicating the follow-up times, and *z *is a vector of covariates representing other variables being adjusted for. (Note that α_1_, α_2_, α_3_, and α_4 _are vectors.) The referent group in this model is the self care group at the first follow-up time. The models will be fitted using GEE to take into account possible correlation within individuals over time. For each follow-up time point that the omnibus *F*-test is statistically significant, we will go on to test whether there is a difference between yoga and self care to address Aim 1 and a difference between yoga and exercise to address Aim 2.

Based on similar studies on this study population we expect to have at least a 90% follow-up rate which reduces the potential for bias due to loss of follow-up. Therefore, our primary analysis will be a complete case analysis including all observed follow-up outcomes, but we will adjust for all baseline covariates that are predictive of outcome, probability of being missing, or differences between treatment groups. We will also conduct sensitivity analyses using an imputation method for non-ignorable non-response to evaluate if our results are robust in the complete case analysis [56]. We will report both results in our manuscript.

To help us further understand the benefits of yoga as a treatment for back pain, we will explore possible interactions between treatment groups and covariates. For example, we will consider interactions of the treatment group and the baseline value that would indicate the effect of treatment depends on status at baseline. We will also test for significant interactions of treatment group with other variables (*e.g*., gender) to determine if treatment differences are modified by these variables. We will use similar methods to analyze secondary outcomes including disability days and satisfaction with care.

If we find that yoga is more effective than self care or exercise we will move to Aim 3 with the goal of exploring whether the beneficial effects of yoga on our primary outcomes are mediated through certain measured variables. As mentioned in Figure 1 and Table 4, we are interested in four major classes of measures that could mediate the effects of yoga on back pain outcomes: (1) physical function, (2) cognitive appraisal, (3) affect and stress, and (4) neuroendocrine function. In the interest of parsimony, we will narrow down the number of perspective variables within the four major classes of proposed mediators by using a modification of the framework described by Baron and Kenney [57].

We will first individually regress each of the potential mediators within a major class on the treatment group. If the potential mediator is associated with the treatment group (α-level = 0.10), we will then evaluate the magnitude of the effect of the individual potential mediator by using an inverse probability weighted (IPW) modeling approach on each of the primary outcomes (i.e., RDQ score or bothersomeness score)[58]. This approach allows us to estimate the direct effect of treatment after rebalancing the treatment groups with respect to the mediator. Specifically, we will first model the probability of the treatment given the mediator using logistic regression. From this model we will obtain the estimated probabilities that each person received their observed treatment given their observed mediator value. We will then use an inverse probability of treatment weighted regression to model the primary outcomes on treatment status while adjusting for the baseline level of the outcome. Comparing the weighted to the unweighted model will allow us to estimate how much of the direct effect of treatment on the outcome can be explained by a potential mediator.

We will do this for all potential mediators in a class and rank them based on how much of the direct effect of treatment on the outcome each explains. The potential mediators within a class that explain at least 10% of the direct effect will then be put in a multiple mediator weighted regression model to assess whether the effect can be mostly explained by a single mediator in the class or if it requires multiple mediators within the class. This stepwise approach will be used to reduce the number of mediators that will be included from a given class. It will not provide estimates of the final strength of the class of mediators since this would require the assumption that classes of potential mediators are independent of one another (i.e., physical function and stress are unrelated). Assessment of the strength of mediation from each class requires that we conduct a multiple mediator analyses evaluating all classes of mediators in a single model [59].

After determining the subset of mediators to be included from each class, we will run a final multiple mediator IPW model. The application of the IPW approach, as compared to the traditional approach of adjusting for multiple mediators, allows us to more appropriately account for confounding between a mediator and the outcome both by additional mediators and by other measured variables [60]. Further we are better able to estimate the indirect effects of each mediator in a causal framework through decomposition of the total effect of treatment into indirect effects through each mediator and the direct effect after accounting for all mediators.

All time points for which there is a significant difference between yoga and self care (or exercise) will be included in the models, and we will use GEE to account for possible correlation within subjects over time.

The results of our previous study suggest that there are several possible scenarios of treatment differences we might expect to find in our proposed trial [19]. Each of these scenarios would result in a different approach to exploring mediating factors. *Scenario 1: *yoga is more effective than self care but not significantly better than exercise, which is not significantly better than self care. In this scenario, we will look for mediators that explain the mechanisms through which yoga works compared to self care, but we cannot explore whether there are different mediators for exercise and yoga if exercise is not significantly better than self care. Thus, we will not be able to determine whether there are mechanisms of healing unique to yoga compared to a "body-focused" treatment like exercise. *Scenario 2: *yoga is significantly better than both self care and exercise, and exercise may or may not be significantly better than self care. In this scenario, we will look for mediators that may explain how yoga works compared to self care and to exercise. It is possible that these mediators are different. By focusing on the yoga vs. exercise difference, we can determine which mediators are most responsible for the unique effects of yoga, which we believe will be mostly those related to increased awareness. *Scenario 3: *both yoga and exercise are better than self care but yoga is not significantly different from exercise. In this scenario, we might conclude that the beneficial effects of yoga are solely or largely due to physical exercise, and that the awareness component has no materially important effect on back pain. However, it is possible that completely different pathways mediate these beneficial effects, and we will be able to determine this through the mediator analysis. For example, the treatment effect of yoga compared to self care might be significantly reduced when a specific mediator variable (e.g., body awareness) is included in the model, with no significant change on the treatment effect of exercise compared to self care.

## List of abbreviations

CAM: Complementary and Alternative Medicine; CATI: computer assisted telephone interviewing; CLIA: Clinical Laboratory Improvement Amendments (certification for US clinical laboratories); CONSORT: CONsolidated Standards of Reporting Trials; DHEA: Dehydroepiandrosterone; GEE: General Estimating Equations; ICD-9: International Classification of Diseases, 9^th ^edition/revision; IPW: Inverse Probability Weighted; IRB: Institutional Review Board; NCCAM: National Center for Complementary and Alternative Medicine; RDQ: modified Roland-Morris Disability Questionnaire; SD: Standard Deviation.

## Competing interests

The authors declare that they have no competing interests.

## Authors' contributions

KJS, DCC, AJC and RAD participated in the conception of the trial and in plans for the analysis of the data. RW participated in plans for the analysis of the data. KJS, DCC, RJH, AJC and RW drafted the manuscript. All authors read and approved the final manuscript.

## References

[B1] Sternbach RA Survey of Pain in the United States: The Nuprin Pain Report Clin J Pain 1986 2 49 53 10.1097/00002508-198602010-00008 3785965

[B2] Frymoyer JW Back pain and sciatica N Engl J Med 1988 318 291 300 2961994 10.1056/NEJM198802043180506

[B3] Martin BI Deyo RA Mirza SK Turner JA Comstock BA Hollingworth W Sullivan SD Expenditures and health status among adults with back and neck problems JAMA 2008 299 656 664 10.1001/jama.299.6.656 18270354

[B4] Stewart WF Ricci JA Chee E Morganstein D Lipton R Lost productive time and cost due to common pain conditions in the US workforce JAMA 2003 290 2443 2454 10.1001/jama.290.18.2443 14612481

[B5] Haldeman S Dagenais S A supermarket approach to the evidence-informed management of chronic low back pain Spine J 2008 8 1 7 10.1016/j.spinee.2007.10.009 18164448

[B6] Consumer Reports How is your doctor treating you? 1995 81 88

[B7] Cherkin DC MacCornack FA Patient evaluations of low back pain care from family physicians and chiropractors West J Med 1989 150 351 355 2525303 PMC1026476

[B8] Greenfield S Anderson H Winickoff RN Morgan A Komaroff AL Nurse-protocol management of low back pain. Outcomes, patient satisfaction and efficiency of primary care West J Med 1975 123 350 359 128907 PMC1129908

[B9] Overman SS Larson JW Dickstein DA Rockey PH Physical therapy care for low back pain. Monitored program of first-contact nonphysician care Phys Ther 1988 68 199 207 2963349 10.1093/ptj/68.2.199

[B10] Carey TS Garrett J Jackman A McLaughlin C Fryer J Smucker DR The outcomes and costs of care for acute low back pain among patients seen by primary care practitioners, chiropractors, and orthopedic surgeons. The North Carolina Back Pain Project N Engl J Med 1995 333 913 917 10.1056/NEJM199510053331406 7666878

[B11] Eisenberg DM Kessler RC Van Rompay MI Kaptchuk TJ Wilkey SA Appel S Davis RB Perceptions about complementary therapies relative to conventional therapies among adults who use both: results from a national survey Ann Intern Med 2001 135 344 351 11529698 10.7326/0003-4819-135-5-200109040-00011

[B12] Wolsko PM Eisenberg DM Davis RB Kessler R Phillips RS Patterns and perceptions of care for treatment of back and neck pain: results of a national survey Spine 2003 28 292 297 10.1097/00007632-200302010-00018 12567035

[B13] Saper RB Eisenberg DM Davis RB Culpepper L Phillips RS Prevalence and patterns of adult yoga use in the United States: results of a national survey Altern Ther Health Med 2004 10 44 49 15055093

[B14] Barnes PM Powell-Griner E McFann K Nahin RL Complementary and alternative medicine use among adults: United States, 2002 Adv Data 2004 1 19 15188733

[B15] Barnes PMBB Nahin R Complementary and Alternative Medicine Use Among Adults and Children: United States, 2007 Natl Health Stat Report 2008 10 1 23 19361005

[B16] Chou R Huffman LH Nonpharmacologic therapies for acute and chronic low back pain: a review of the evidence for an American Pain Society/American College of Physicians clinical practice guideline Ann Intern Med 2007 147 492 504 17909210 10.7326/0003-4819-147-7-200710020-00007

[B17] Galantino ML Bzdewka TM Eissler-Russo JL Holbrook ML Mogck EP Geigle P Farrar JT The impact of modified Hatha yoga on chronic low back pain: a pilot study Altern Ther Health Med 2004 10 56 59 15055095

[B18] Williams KA Petronis J Smith D Goodrich D Wu J Ravi N Doyle EJ Jr Gregory Juckett R Munoz Kolar M Gross R Steinberg L Effect of Iyengar yoga therapy for chronic low back pain Pain 2005 115 107 117 10.1016/j.pain.2005.02.016 15836974

[B19] Sherman KJ Cherkin DC Erro J Miglioretti DL Deyo RA Comparing yoga, exercise, and a self-care book for chronic low back pain: a randomized, controlled trial Ann Intern Med 2005 143 849 856 16365466 10.7326/0003-4819-143-12-200512200-00003

[B20] Tekur P Singphow C Nagendra HR Raghuram N Effect of short-term intensive yoga program on pain, functional disability and spinal flexibility in chronic low back pain: a randomized control study J Altern Complement Med 2008 14 637 644 10.1089/acm.2007.0815 18673078

[B21] Groessl EJ Weingart KR Aschbacher K Pada L Baxi S Yoga for veterans with chronic low-back pain J Altern Complement Med 2008 14 1123 1129 10.1089/acm.2008.0020 18991515

[B22] Williams K Abildso C Steinberg L Doyle E Epstein B Smith D Hobbs G Gross R Kelley G Cooper L Evaluation of the effectiveness and efficacy of iyengar yoga therapy on chronic low back pain Spine (Phila Pa 1976) 2009 34 2066 2076 19701112 10.1097/BRS.0b013e3181b315cc PMC4393557

[B23] Kroenke K Spitzer RL Williams JB The PHQ-9: validity of a brief depression severity measure J Gen Intern Med 2001 16 606 613 10.1046/j.1525-1497.2001.016009606.x 11556941 PMC1495268

[B24] Moore JE Lorig K Von Korff M Gonzalez V Laurent D The Backpain Helpbook 1999 Reading, MA, Perseus Publishing

[B25] Kraftsow G Yoga for Wellness 1999 New York: Arkana

[B26] Bombardier C Outcome assessments in the evaluation of treatment of spinal disorders: summary and general recommendations Spine 2000 25 3100 3103 10.1097/00007632-200012150-00003 11124724

[B27] Patrick DL Deyo RA Atlas SJ Singer DE Chapin A Keller RB Assessing health-related quality of life in patients with sciatica Spine 1995 20 1899 1908 10.1097/00007632-199509000-00011 8560339

[B28] Roland M Fairbank J The Roland-Morris Disability Questionnaire and the Oswestry Disability Questionnaire Spine 2000 25 3115 3124 10.1097/00007632-200012150-00006 11124727

[B29] Deyo RA Diehl AK Measuring physical and psychosocial function in patients with low-back pain Spine 1983 8 635 642 10.1097/00007632-198309000-00009 6228020

[B30] Deyo RA Early diagnostic evaluation of low back pain J Gen Intern Med 1986 1 328 338 10.1007/BF02596214 2945917

[B31] Deyo RA Centor RM Assessing the responsiveness of functional scales to clinical change: an analogy to diagnostic test performance J Chronic Dis 1986 39 897 906 10.1016/0021-9681(86)90038-X 2947907

[B32] Roland M Morris R A study of the natural history of back pain. Part I: development of a reliable and sensitive measure of disability in low-back pain Spine 1983 8 141 144 10.1097/00007632-198303000-00004 6222486

[B33] Roland M Morris R A study of the natural history of back pain. Part II: development of guidelines for trials of treatment in primary care Spine 1983 8 145 150 10.1097/00007632-198303000-00005 6222487

[B34] Deyo RA Measuring the functional status of patients with low back pain Arch Phys Med Rehabil 1988 69 1044 1053 2975164

[B35] Cherkin DC Deyo RA Battie M Street J Barlow W A comparison of physical therapy, chiropractic manipulation, and provision of an educational booklet for the treatment of patients with low back pain N Engl J Med 1998 339 1021 1029 10.1056/NEJM199810083391502 9761803

[B36] Cherkin DC Eisenberg D Sherman KJ Barlow W Kaptchuk TJ Street J Deyo RA Randomized trial comparing traditional Chinese medical acupuncture, therapeutic massage, and self-care education for chronic low back pain Arch Intern Med 2001 161 1081 1088 10.1001/archinte.161.8.1081 11322842

[B37] Dunn KM Croft PR DUP - Classification of low back pain in primary care: using "bothersomeness" to identify the most severe cases Spine 2005 30 1887 1892 10.1097/01.brs.0000173900.46863.02 16103861

[B38] Vlaeyen JW Kole-Snijders AM Boeren RG van Eek H Fear of movement/(re)injury in chronic low back pain and its relation to behavioral performance Pain 1995 62 363 372 10.1016/0304-3959(94)00279-N 8657437

[B39] Von Korff M Moore JE Lorig K Cherkin DC Saunders K Gonzalez VM Laurent D Rutter C Comite F A randomized trial of a lay person-led self-management group intervention for back pain patients in primary care Spine 1998 23 2608 2615 10.1097/00007632-199812010-00016 9854760

[B40] Lorig K Chastain RL Ung E Shoor S Holman HR Development and evaluation of a scale to measure perceived self-efficacy in people with arthritis Arthritis Rheum 1989 32 37 44 10.1002/anr.1780320107 2912463

[B41] Shields S Mallory M Simon A The body awareness questionnaire: reliability and validity J Personality Assessment 1989 53 802 815 10.1207/s15327752jpa5304_16

[B42] Daubenmier J The relationship of yoga, body awareness, and body responsiveness to self-objectification and disordered eating Psychology of Women Quarterly 2005 29 207 219 10.1111/j.1471-6402.2005.00183.x

[B43] Ware JE Jr Sherbourne CD The MOS 36-item short-form health survey (SF-36). I. Conceptual framework and item selection Med Care 1992 30 473 483 10.1097/00005650-199206000-00002 1593914

[B44] Ware J Kosinski M SK K SF-36 phyical and mental health summary scales: A user's manual 1994 Boston, MA, The Health Institute

[B45] Cohen S Williamson G Spacapan S, Oskamp S Perceived stress in a probability sample of the United States The social psychology of health: Claremont Symposium on applied social psychology 1988 Newbury Park, CA: Sage

[B46] Adler NE Horowitz M Garcia A Moyer A Additional validation of a scale to assess positive states of mind Psychosom Med 1998 60 26 32 9492235 10.1097/00006842-199801000-00006

[B47] Waddell G Somerville D Henderson I Newton M Objective clinical evaluation of physical impairment in chronic low back pain Spine 1992 17 617 628 10.1097/00007632-199206000-00001 1308095

[B48] Kirschbaum C Hellhammer DH Salivary cortisol in psychobiological research: an overview Neuropsychobiology 1989 22 150 169 10.1159/000118611 2485862

[B49] Granger DA Schwartz EB Booth A Curran M Zakaria D Assessing dehydroepiandrosterone in saliva: a simple radioimmunoassay for use in studies of children, adolescents and adults Psychoneuroendocrinology 1999 24 567 579 10.1016/S0306-4530(99)00013-X 10378242

[B50] Altman D Shulz K Moher D Egger M Davidoff F Elbourne D Getzche P Lang T The revised CONSORT statement for reporting clinical trials: explanation and elaboration Ann Intern Med 2001 134 663 694 11304107 10.7326/0003-4819-134-8-200104170-00012

[B51] Levin J Serlin R Seaman M A controlled, powerful multiple-comparison strategy for several situaitons Psychological Bulletin 1994 115 153 159 10.1037/0033-2909.115.1.153

[B52] Rothman K Greenland S Modern Epidemiology 1998 Philadelphia: Lippincott-Raven

[B53] Rothman KJ No adjustments are needed for multiple comparisons Epidemiology 1990 1 43 46 2081237

[B54] Savitz DA Olshan AF Multiple comparisons and related issues in the interpretation of epidemiologic data Am J Epidemiol 1995 142 904 908 7572970 10.1093/oxfordjournals.aje.a117737

[B55] Zeger SL Liang KY Longitudinal data analysis for discrete and continuous outcomes Biometrics 1986 42 121 130 10.2307/2531248 3719049

[B56] Wang M Fitzmaurice GM A simple imputation method for longitudinal studies with non-ignorable non-responses Biom J 2006 48 302 318 10.1002/bimj.200510188 16708780

[B57] Baron RM Kenny DA The moderator-mediator variable distinction in social psychological research: conceptual, strategic, and statistical considerations J Pers Soc Psychol 1986 51 1173 1182 10.1037/0022-3514.51.6.1173 3806354

[B58] Cole SR Hernan MA Constructing inverse probability weights for marginal structural models Am J Epidemiol 2008 168 6 656 664 10.1093/aje/kwn164 18682488 PMC2732954

[B59] Preacher KJ Hayes AF Asymptotic and resampling strategies for assessing and comparing indirect effects in multiple mediator models Behav Res Methods 2008 40 3 879 891 10.3758/BRM.40.3.879 18697684

[B60] VanderWeele TJ Marginal structural models for the estimation of direct and indirect effects Epidemiology 2009 20 1 18 26 10.1097/EDE.0b013e31818f69ce 19234398

